# A Systematic Review on the Role of Matrix Metalloproteinases in the Pathogenesis of Inguinal Hernias

**DOI:** 10.3390/biom13071123

**Published:** 2023-07-14

**Authors:** Umberto Bracale, Roberto Peltrini, Biancamaria Iacone, Mirko Martirani, Daniele Sannino, Antonio Gargiulo, Francesco Corcione, Raffaele Serra, Umberto Marcello Bracale

**Affiliations:** 1 Department of Advanced Biomedical Sciences, University of Naples Federico II, 80131 Naples, Italy; 2 Department of Public Health, University of Naples Federico II, 80131 Naples, Italy; 3 Department of Medical and Surgical Sciences, University Magna Graecia of Catanzaro, 88100 Catanzaro, Italy

**Keywords:** matrix metalloproteinases, inguinal hernia, collagen, extracellular matrix, biomarkers

## Abstract

The recurrence rate in patients who undergo surgery for abdominal wall hernias (AWHs) is high. AWHs have been hypothesized to be a disease of the extracellular matrix, which is supported by evidence showing a high incidence of AWHs in patients with connective tissue disorders. This study aimed to investigate the most recent literature studies describing the levels of several matrix metalloproteinases (MMPs) in the blood and fascia, with the objective of better clarifying the pathogenetic role of matrix metalloproteinases (MMPs) and their inhibitors in inguinal hernias (IHs). A systematic literature search was conducted using the PubMed, Scopus, and Web of Science electronic databases to identify eligible studies. The identified studies were included in the analysis, and a qualitative synthesis of the results is provided to describe the most recent findings. Seventeen studies were included. An association between MMP-2 and direct IHs has also been demonstrated. MMP-1, MMP-2, MMP-9, MMP-12, and MMP-13 levels were increased in both the serum and fascia of patients with IHs. The analysis of inhibitors showed an increase in tissue inhibitors of metalloproteinases (TIMPs), specifically TIMP-1 in IHs, particularly in direct hernias, and a reduction in TIMP-2 in the biopsy samples of the transversalis fascia. In contrast, a reduction in TIMP-1 and an increase in TIMP-2 levels have been reported only in the serum of patients with IHs. Metalloproteinases play a crucial role in the pathogenesis of IHs. The analysis of other molecules, such as TIMPs or their correlation with specific genes, is enhancing our understanding of the pathophysiology of IHs. However, more prospective studies, including comprehensive clinical and laboratory data collection, are required to confirm the relationship between the studied biomarkers and the risk of IHs.

## 1. Introduction

Abdominal wall hernias (AWHs) and groin hernias are common indications for surgery worldwide. While primary hernias are considered to be the manifestation of a congenital condition that allows hernia formation in childhood or adulthood, incisional hernias occur in approximately 10–30% of patients who undergo open surgeries via laparotomy, depending on several risk factors [1]. Despite the use of different prosthetic materials, techniques, and approaches (a “tailored approach”) [2], recurrence rates remain unacceptably high [3]. The failure of surgical techniques to yield satisfactory outcomes has prompted researchers to investigate the pathophysiology of the disease. Therefore, disorders of collagen metabolism have been proposed to be associated with AWHs and their high recurrence rates. The hypothesis that AWHs represent a disease of the extracellular matrix (ECM) is supported by evidence of a high incidence of AWH in patients with connective tissue disorders, such as Ehlers-Danlos and Marfan syndrome [4,5]. Collagen, the primary protein in the ECM, contributes to its tensile strength. There are 28 different types of collagens, with two being significant in inguinal hernias (IHs): type I, which represents the mature and most stable form of collagen, and type III collagen, an immature isoform that is present at a higher concentration in the ECM of patients with AWHs [6,7].

There are two causes for the decreased type I/type III collagen ratio in the connective tissue of patients with AWHs: either a primary defect in collagen synthesis or the altered collagen expression caused by excessive degradation of the extracellular matrix (ECM). Matrix metalloproteinases (MMPs) are the key enzymes involved in ECM degradation [8]. It is now evident that the collagenolytic activity of MMPs plays a role in the pathophysiology of AWHs. The disrupted collagen type I/type III ratio in the fascia of AWH patients is attributed to altered collagen degradation resulting from overexpression of MMPs. The association between MMP overexpression and AWHs was initially demonstrated by Bellon et al., who investigated the expression of MMP-1 and MMP-2 in the transversalis fascia of patients with primary and recurrent IHs [9], suggesting different pathophysiological mechanisms of inguinal herniation for patients with direct and indirect IHs. Subsequent research has focused on exploring the role of MMPs in AWH development. The aim of this systematic review is to provide an exhaustive overview of the different pathophysiological mechanisms in AWHs by highlighting the role of MMPs.

## 2. Materials and Methods

We conducted a systematic review following the Preferred Reporting Items for Systematic Reviews and Meta-Analyses (PRISMA) statement [10]. The review focused on the most recent evidence available up to 2009 [11]. We searched the PubMed, Scopus, and Web of Science databases from January 2010 to December 2022. The inclusion criteria for this study included relevant cohorts, case series, and cross-sectional studies that examined the relationship between abnormal MMP expression and primary inguinal hernia. The following search algorithm was used: ((“metalloproteases”[MeSH Terms] OR “metalloproteases”[All Fields] OR “metalloproteinase”[All Fields] OR “metalloproteinases”[All Fields] OR “metalloproteinase s”[All Fields]) AND (“hernia, inguinal”[MeSH Terms] OR (“hernia”[All Fields] AND “inguinal”[All Fields]) OR “inguinal hernia”[All Fields] OR (“inguinal”[All Fields] AND “hernia”[All Fields]) OR ((“groin”[MeSH Terms] OR “groin”[All Fields] OR “groins”[All Fields]) AND (“hernia”[MeSH Terms] OR “hernia”[All Fields] OR “hernias”[All Fields] OR “hernia s”[All Fields] OR “herniae”[All Fields])))) AND (2010:2022[pdat]).

Duplicate studies and those that did not meet the eligibility criteria, such as review articles or studies involving incisional or recurrent hernias, were excluded.

Two authors (RP and BI) independently assessed the titles and abstracts of the identified studies, and the full published reports of potentially eligible studies were thoroughly reviewed. Any disagreements were resolved through consensus. Only studies written in English and including more than 10 patients, which investigated the association between MMP overexpression and inguinal hernia, were included. The included studies provided evidence based on accredited laboratory tests.

Data extraction and outcomes

The data recorded for each eligible study included the year of publication, type of sample, type of hernia, the total number of patients, number of controls, division of patients into subgroups, substances measured, and the method used.

A qualitative synthesis of the results was performed to identify any potential new evidence on the relationship between MMPs and inguinal hernias.

## 3. Results

**Figure 1:**
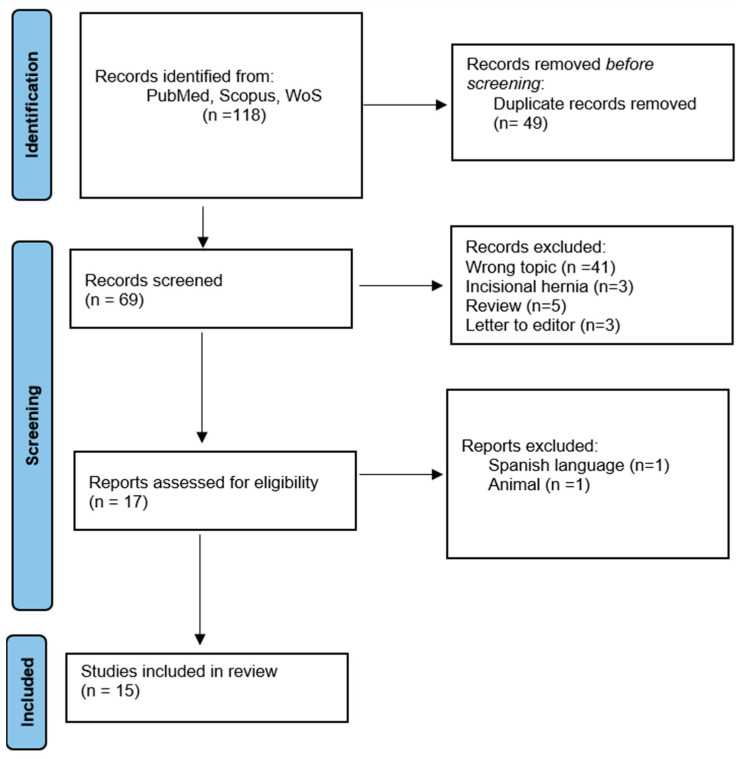
PRISMA flow diagram for systematic review including databases search.

A total of 118 studies were found in the literature. Duplicate studies or those that did not meet eligibility criteria, such as review articles or studies that included incisional or recurrent hernias, were excluded (n = 101). The full texts of 17 studies were evaluated and two of them were excluded because they were written in Spanish and included lab rats in the study. We selected 15 studies, and data regarding IH were extracted from all included studies (Figure 1).

The details of the included studies are presented in Table 1. Eleven studies [12,13,14,15,16,17,18,19,20,21] focused only on IHs and compared them to a control group. Four studies included patients with IHs and other diseases. In 2011, Antoniou et al. [22] compared two groups: thirty-three patients with abdominal aortic aneurysm (AAA), a group of 95 patients with IHs, and a control group of 35 patients. Three other studies [17,20,23] focused on metalloproteinase assays (MMP-1, 2, 9, 12, 13). Jain et al. [24] reported only on MMP-2, while other studies investigated the levels of other modulators such as tissue inhibitors of metalloproteinases (TIMPs), TIMP-1, TIMP-2, P1NP, P3NP, ELN, and gene expression of EFEMP1, COL1A1, and COL3A, which are components of ECM, in addition to metalloproteinases.

### 3.1. MMPs

**Figure 2:**
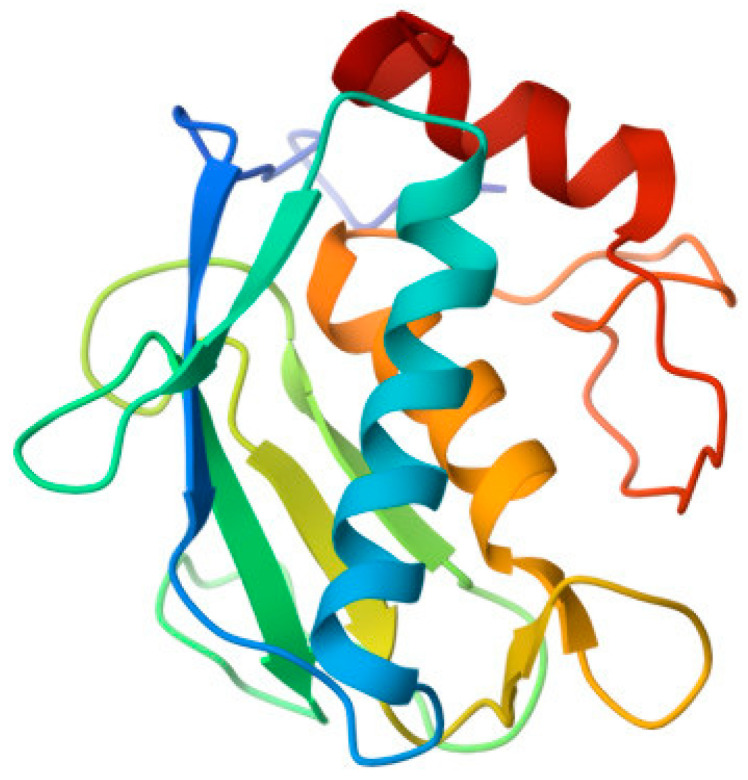
Crystal structure of the human MMP-2 catalytic domain in a complex with an inhibitor. From the RCSB Protein Data Bank (https://www.rcsb.org/, accessed on 22 June 2023).

MMPs, a subgroup of zinc metalloproteinases, serve important functional roles in regulating cell growth, apoptosis, angiogenesis, and the immune system. Their involvement in the degradation of the extracellular matrix (ECM) is crucial, as the ECM is produced by inflammatory and mesenchymal cells. The activity of MMPs is tightly regulated through mechanisms such as gene expression control, zymogen activation, and inhibition by specific inhibitors, predominantly α-2-macroglobulin and tissue inhibitors of MMPs (TIMPs) [11]. MMPs have been implicated in the pathophysiology of AWHs primarily due to their collagenolytic activity. The overexpression of MMPs leads to an imbalance in the ratio of type I to type III collagen in patients with inguinal hernias. MMPs exhibit structural similarities and are composed of multiple domains. All members of the family contain a pre-domain involved in enzyme secretion, an autoinhibitory pro-domain, a catalytic domain responsible for enzyme activity, and a hemopexin-like domain involved in substrate recognition. MMP-2 and MMP-9 also have a fibronectin-like sequence in their catalytic domain. All MMP pro-domains consistently feature a cysteine residue that binds zinc and activates the enzymes. The activated forms are subsequently released when the cysteine dissociates from the catalytic zinc, following the “cysteine switch” mechanism. Specifically, MMP-2 (Figure 2) is also known as gelatinase A and is located on chromosome 16q13. It is responsible for cleaving collagen types I, II, III, IV, V, and IX [25,27].

The available evidence suggests that MMP-2 is the most important metalloproteinase in the pathophysiology of inguinal hernias, particularly in direct inguinal hernias (IHs), as highlighted in the review by Antoniou et al. [11] and confirmed in the 11 articles we evaluated. Jain et al. [24] demonstrated an increase in MMP-2 levels in the blood plasma and transversalis fascia of patients with direct IHs. In contrast, Antoniou et al. [22] found significantly lower MMP-2 levels in the blood plasma of 91 patients with hernias compared to patients without hernias. However, this study did not differentiate the patients according to the inguinal hernia subtype. Aren et al. [16] observed an increase in MMP-1, MMP-2, and MMP-9 levels in the transversalis fascia of patients with direct and indirect IHs, as compared to controls. Serra et al. [23], who evaluated the levels of MMP-1, MMP-2, MMP-9, MMP-12, and MMP-13 in tissue and plasma samples using ELISA, reported a significant increase in all patients affected by IHs compared to controls. Li et al. [12] observed a significant increase in MMP-2 levels in both the direct and recurrent IH groups, along with a significant decrease in the ratio of P1NP/P3NP. Pascual et al. [18] investigated the expression of MMP-2 and its modulators MT1-MMP and TIMP-2 in skin fibroblasts using specific antibodies. They found significantly higher expression of active MMP-2 in patients with direct IHs, and the expression of MT1-MMP was directly correlated with MMP-2 expression, showing the most intense staining in patients with direct and indirect IHs. Additionally, TIMP-2 exhibited maximal expression in the control group while the hernia group displayed significantly decreased expression levels. MMP-2, MMP-7, MMP-10, and MMP-12 are elastolytic metalloproteinases. MMP-12 may indirectly affect elastolysis through the digestion of chemokines and other extracellular proteins which are physiological substrates of MMP-12. Structure-function studies of elastolysis have focused on MMP-12, MMP-2, and MMP-9, and have mapped extensive binding sites for elastin. MMP-2 and MMP-9 require their fibronectin-like modules to bind and digest elastin [28].

Strohalmová et al. [20] conducted a study comparing pre- and post-operative serum levels of MMP-1, MMP-2, MMP-7, MMP-9, MMP-10, TIMP-1, and TIMP-2 in patients with direct and indirect His who underwent surgery. They employed ELISA as the method of analysis and observed a decrease in the levels of most MMPs, except for MMP-9, which showed a significant increase. Notably, the most significant decrease was observed for MMP-2. Additionally, they found that levels of TIMP-1 and TIMP-2 significantly decreased after surgery, while the ratio of MMP-9 to TIMP-1 significantly increased. MMP-1 and MMP-13 belong to the collagenase group of MMPs. These genes are located on chromosome 11q22-q23 and their primary function is to cleave collagen types I, II, and III [29]. The involvement of MMP-2 in the pathogenesis of direct IHs is supported by Antoniou et al. [11]. Moreover, it appears evident that MMP-1 and MMP-13 are likely implicated in the pathophysiology of recurrent IHs, as indicated by studies [23,30,31]. Among the studies we analysed, four of them also measured the levels of MMP-1 and MMP-13. Serra et al. [23] discovered significantly elevated levels (*p* < 0.01) of MMP-1 and MMP-13 in patients with IHs compared to those without IHs. Aren et al. [16] reported higher levels of MMP-1 and other MMPs (MMP-2 and MMP-9) in the transversalis fascia of 60 patients with direct and indirect IHs. Isik et al. [17] found increased levels of MMP-1 and MMP-13 in 44 patients with direct and indirect IHs, with higher levels observed in patients with bilateral IHs. Strohalmová et al. [20] reported a non-significant (*p* = 0.016) decrease in MMP-1 levels in the post-operative period of patients who underwent surgery for IHs. MMP-9, which is in the 20q11.2-q13.1 genomic region, is a gelatinase responsible for cleaving elastin and type IV collagen [23]. Four studies confirmed the overexpression of MMP-9 in IH patients. In two of these studies, overexpression of MMP-9 was observed in both serum and tissue, while the other two studies reported an increase specifically in the transversalis fascia tissues. On the contrary, three studies [15,20,22] reported a significantly lower concentration of MMP-9 in the blood plasma of IH patients.

### 3.2. TIMPs

**Figure 3:**
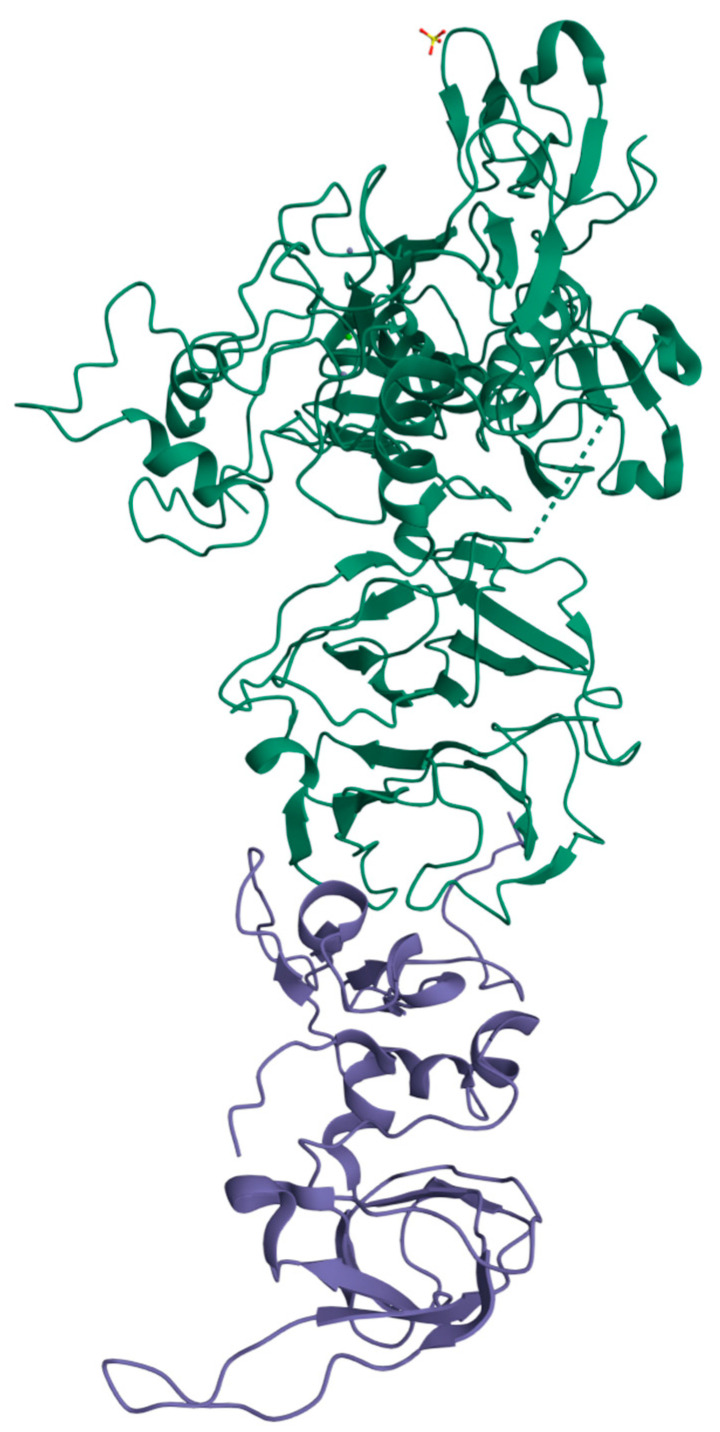
proMMP-2/TIMP-2 complex. From the RCSB Protein Data Bank (https://www.rcsb.org/, accessed on 22 June 2023).

Tissue inhibitors of metalloproteinases (TIMPs) are responsible for preventing excessive degradation of the extracellular matrix. The gene TIMP1, located on chromosome 22q12.3, encodes TIMP-1. TIMP-1 acts as an inhibitor of MMPs and plays a crucial role in maintaining the homeostasis of the extracellular matrix (ECM). TIMPs have an N-terminal domain that folds as a separate unit and is capable of inhibiting MMPs. The molecule has a wedge-shaped structure that inserts into the active-site cleft of an MMP. Most TIMPs exhibit a characteristic N-terminal sequence, C-X-C, in which an amino acid (threonine or serine in vertebrates) is situated between the first and second cysteines. The function of this conserved sequence is to interact with the S1 pocket, a section of MMPs [26]. TIMP-2 has several distinct properties and functions. These properties include inhibition of tyrosine kinase receptor signalling by binding to α3β1 integrin on the cell surface and activation of Shp-1 protein tyrosine phosphatase activity [32]. TIMP-2 is located on human chromosome 17q25.3 [11]. TIMP-2 has an activating action on MMP-2. Pro-MMP-2 forms a complex with TIMP-2 through interactions between the hemopexin-like domain of MMP-2 and the non-inhibitory C-terminal domain of TIMP-2 (Figure 3). Subsequently, the complex is activated by MT1-MMP. On the cell surface, it binds via the free inhibitory N-terminal domain of TIMP-2 along with a second MT1-MMP, generating a tetramer. One MT1-MMP acts as a receptor for the Pro-MMP-2-TIMP-2 complex and the other one as an activator of pro-MMP-2. Excess TIMP-2 prevents this activation process [27]. The roles of TIMP-1 and TIMP-2 in the context of inguinal hernias (IHs) have been extensively studied. Durukan et al. [31] demonstrated higher immunohistochemical expression of TIMP-1 and reduced expression of TIMP-2 in transversalis fascia samples from patients with direct IHs. On the other hand, Isik et al. [17] evaluated the expression of TIMP-1, TIMP-2, and TIMP-3 in serum and transversalis fascia. They found progressively lower expression of TIMPs in patients with IHs, with a drastic decrease in patients with bilateral IHs compared to the control group. In contrast, Antoniou et al. [15] found low levels of TIMP-1 and high levels of TIMP-2 in blood plasma and abdominal fascia tissue samples. However, there was a lower expression of both TIMP-1 and TIMP-2 in the tissue of patients with IHs when compared to controls. Wang et al. [13] measured MMP-2 mRNA and TIMP-2 mRNA expression on the anterior rectus sheath and reported an increase in MMP-2 mRNA levels in both direct and indirect IH patients when compared to the controls. They also observed a reduction of TIMP-2 mRNA levels in patients with direct IHs, resulting in a higher ratio between the two values in both groups of patients with IHs. One year later, Wang et al. [13] investigated the correlation between MMP-2 and TIMP-2 expression and patient age. They included all patients with a primary inguinal hernia and divided them into study groups based on age. They measured mRNA for both groups in the anterior rectus sheath. They found that older patients with a primary IH exhibited increased expression of MMP-2 and reduced expression of TIMP-2 compared to the control group. In a study by Smigielski et al. [14], they also assessed the expression of MMP-2 and TIMP-2 in the serum of patients with direct, indirect, or recurrent IHs, dividing them into subgroups based on age. The results showed that MMP-2 levels were higher in all subgroups of younger patients, especially in those with direct IHs. On the other hand, TIMP-2 levels in the subgroup of younger men were statistically higher than those in older patients, particularly in those with recurrent IHs. In their study, Strohalmová et al. [20] conducted a comparison of pre-and post-operative serum levels of MMP-1, MMP-2, MMP-7, MMP-9, MMP-10, TIMP-1, and TIMP-2 in patients who underwent surgery for direct and indirect inguinal hernias. ELISA was employed as the method to measure these levels. The results indicated a decrease in MMP levels, except for MMP-9, which exhibited a significant increase. Notably, the most substantial decrease was observed for MMP-2. Furthermore, TIMP-1 and TIMP-2 levels demonstrated a significant decrease following the surgical procedure. Additionally, the ratio of MMP-9 to TIMP-1 showed a significant increase after surgery. Pascual et al. [18] found MMP-2 expression, together with its modulators (MT1-MMP and TIMP-2), in examined skin fibroblasts. The results revealed significantly higher levels of active MMP-2 expression in patients with direct inguinal hernias. The expression of MT1-MMP was directly correlated with MMP-2 expression, and the most intense staining was observed in patients with an inguinal hernia. On the other hand, TIMP-2 exhibited high expression levels in the control group, while significantly lower expression levels were recorded in the hernia group. In a study conducted by Nizar et al. [21], the expression of COL1A1, COL3A1, and MMP-mRNA was assessed. The findings revealed that COL3A1 showed higher expression in patients with inguinal hernias (IHs), while the expression of COL1A1 in IHs was lower, although this difference did not reach statistical significance. Moreover, a significant positive correlation was observed between the expression of MMP-2, COL1A1, and COL3A1 in patients with IHs. Peng et al. [20] conducted a study to investigate factors associated with the metabolism of collagen and elastin to gain insight into the pathogenesis of inguinal hernias (IHs). They examined the expression of EFEMP1, TIMP-3, MMP-9, and elastin (ELN) in the fascia transversalis of patients with IHs, comparing them to a control group. They discovered that patients with direct IHs exhibited decreased expression of EFEMP1, TIMP3, and ELN, while MMP-9 levels were increased compared to the control group. Additionally, they observed a positive correlation between EFEMP1 and ELN expression, as well as between TIMP3 and ELN expression.

## 4. Discussion

This review aimed to provide a clearer understanding of the involvement of ECM remodelling and collagen turnover in the development of IHs. Specifically, we focused on examining the role of MMPs, TIMPs, and markers associated with collagen turnover and ECM remodelling, as they have been identified as potential biomarkers through comparisons between IH patients and individuals without hernias. The identification of biological markers that can indicate an individual’s genetic predisposition to IHs can be highly beneficial. By detecting these alterations before surgery, surgeons can make more informed decisions regarding the appropriate treatment strategy and the selection of mesh prosthesis for a specific case. The MMP family is an essential group of extracellular proteinases. Their role has been evaluated in various physiological and pathological processes. These enzymes play a critical role in ECM turnover, tissue remodeling, angiogenesis, and morphogenesis. They are also involved in cell migration, invasion, proliferation, and apoptosis. The activity of MMPs is regulated through gene expression, control of zymogens, and inhibition by specific inhibitors such as α-2 macroglobulin and TIMPs. The inhibitor activity of TIMPs is the result of noncovalent binding with MMPs. Specifically, the interaction between TIMPs and MMPs is exerted through the formation of a transmembrane complex with two MT1-MMPs, leading to the activation and degradation of ECM components, promoting the pathogenesis of inguinal hernias [25,31].

The available evidence strongly suggests that metalloproteinases play a crucial role in the pathogenesis of IHs. Specifically, a significant association between MMP-2 and direct inguinal hernias has been demonstrated. However, Antoniou et al. [11] found insufficient or controversial data regarding the association between IH and MMP1, MMP13, and MMP-9 when analysing the available studies. Over time, the literature has been enriched with new studies that not only investigate this group of MMPs but also examine TIMPs and gene expression, which have been included in our updated review. Among the included articles, new associations regarding TIMPs have emerged, providing valuable insights into their role in the development of an inguinal hernia. The extracted data reveal significant controversy, with some studies suggesting an increase in TIMP-1 levels in IHs, specifically in direct hernias, while others report a decrease in TIMP-2 levels in biopsy samples of the transversalis fascia [31]. In contrast, previous studies have reported a decrease in TIMP-1 levels and an increase in TIMP-2 levels specifically in the serum of patients with inguinal hernias [22]. On the other hand, there is a larger body of evidence supporting the role of MMPs (matrix metalloproteinases) in inguinal hernias, as multiple articles have consistently shown increased levels of MMP-1, 2, 9, 12, and 13 in both the serum and fascia of patients with inguinal hernias [16,17,23]. Recent studies have provided new evidence regarding the expression of the genes COL3A1, COL1A1, ELN, and EFEMP-1 in tissues using RT-PCR [19,21]. The studies we analysed were a pool of both observational and prospective studies; however, there were confounding factors and biases. More prospective studies that include extensive clinical and laboratory data collection are needed to confirm the hypothesized relationships between the biomarkers studied and IH risk.

## 5. Conclusions

Recognizing a panel of biomarkers with specific predictive values for the risk of developing inguinal hernias (IHs) in the general population holds the potential to identify high-risk individuals, implement preventive measures, and guide appropriate treatment selection. To achieve this, prospective studies involving hernia-free populations and populations stratified by hernia subtype, as well as standardization of the biomarker detection methods, will be crucial.

## Figures and Tables

**Table 1:** Details of the included studies. [Increase (↑) Decrease (↓)].

Ref./Year	Type of Hernia	Type of Sample	Pat./Ctrl. (n)	Groups Studied (n Patients)	Substances Measured	Method Used	Results
[11], 2011	DINGH, IINGH	Blood plasma, abdominal fascia tissue	126/35	INGH (91), controls (35)	MMP2, MMP9, TIMP1, TIMP2	ELISA	MMP-9 AND MMP-2 in serum are ↓ in INGH then controls; TIMP-1 in serum is ↓ in INGH then controls; TIMP-2 in serum is ↑ in INGH then controls; MMP-9 and MMP-2 in tissue are ↑ in INGH then controls; TIMP-1 and TIMP-2 in tissue are ↓ in INGH then controls.
[12], 2018	DINGH, IINGH, RDINGH, RIINGH	Blood serum	155/45	DINGH (40); RDINGH (10); IINGH (45); RIINGH (15); controls (45)	MMP2; MMP9	ELISA; spectrometer	MMP2 ↑ in RDINGH, RIINGH and DINGH; MMP9 no difference.
[13], 2020	DINGH, IINGH	Anterior rectus sheath fascia	58/10	DIINGH (16); IINGH (32); controls (10)	MMP2 mRNA; TIMP2 mRNA	RT-PCR; Immunohistochemistry	MMP2 ↑ in DINGH/IINGH; TIMP2 ↓ in DINGH; Ratio of MMP2 mRNA/TIMP2 mRNA ↑ in DINGH/IINGH
[14], 2011	DINGH, IINGH, RINGH	Blood serum	150/30	DINGH; IINGH; RINGH; Control; subgroup A: age 26–49 B: age 50–70	MMP2; TIMP2;	ELISA	MMP2 ↑ in DINGH-A, IINGH-A, RINGH A; TIMP2 ↑ in RINGH
[16], 2011	DIINGH, IINGH	Fascia trasversalis	90/30	DIINGH (30); IINGH (30); Controls (30)	MMP-1, MMP-2, MMP-9	Immunohistochemical	↑ MMP-1, -2, -9 in DINGH and IINGH
[17], 2017	IINGH, DINGH, BINGH	Serum and FT	44/11	DINGH (11), IINGH (11), BINGH (11), controls (11)	MMP-1, MMP-2, MMP-9, MMP-13, TIMP-1, TIMP-2, TIMP3.	ELISA	↑ MMP-1, -2, -9, -13 in DINGH, IINGH; ↑↑ in BINGH; TIMPs ↓ in every group then controls; TIMPs ↓↓ in BINGH
[18], 2021	Primary inguinal hernia	Anterior rectus sheath fascia	48/8	Primary: Group A (50–59 aa) (10); Group B (60–69 aa) (10); Group C (70–79 aa) (10); Group D (80–89 aa) (10) Controls (8)	MMP-2, TIMP-2	RT-PCR	MMP-2 ↑ in Group C and D then controls; TIMP-2 is ↓ in Group C and D then controls; positive correlation between age and MMP-s levels; negative correlation between age and TIMP-2 levels
[19], 2010	DINGH, IINGH, CONTROLS	Skin biopsy	46/10	Group I (median age 43) DINGH (18), Group II (median age 39,7) IINGH (18), Controls (median age 39,5) (10)	MMP-2, MT-1 MMP, TIMP-2	Immunocytochemistry, Immunoblotting, Zymography	MMP-2 expression in skin fibroblasts is ↑ in DINGH; positive correlation between MT1-MMP expression and MMP-2 expression in DINGH AND IINGH; TIMP-2 ↑ IN CONTROL GROUP
[20], 2020	DINGH, CONTROLS	FT	40/20	DINGH (20), CONTROLS (20)	TIMP-3, MMP-9	Immunohistochemical, western blot, RT-PCR	TIMP-3 ↓ in DINGH then Controls; MMP-9 level ↑ in DINGH then controls. Negative correlation between TIMP3 and MMP9
[21], 2021	DINGH, IINGH, RINGH, controls	Blood serum	46/21	DINGH (8), INGH (10), RINGH (4), CONTROLS (21) pre and post operative (24 h after the surgery)	MMP-1, MMP-2, MMP-7, MMP-9, MMP-10, TIMP-1, TIMP-2	ELISA	preoperative: MMP-2 MMP-9 ↓ in INGH, MMP-1,-7,-10 no difference; TIMP-2 ↓ in INGH; TIMP-1 no difference; MMP-9/TIMP-1 ↓ in INGH; postoperative: MMPs significant ↓ except MMP-9 that ↑ in IH; MMP-9/TIMP-1 ↑ in IH; not observe differences in MMPs/TIMPs in subgroups of IH
[22], 2021	INGH, CONTROLS	Aponeurosis Musculus obliquus externus		INGH (50.92 ± 13.09 years), CONTROLS (36.16 ± 12.53)	MMP-2 mRNA	RT-PCR	MMP2 expression higher in INGH
[23], 2010	AAA, INGH, CONTROLS	Blood plasma	159/35	AAA (33), INGH (91), CONTROLS (35)	MMP-2, MMP-9, TIMP1, TIMP2	ELISA	MMP-9 and MMP-2 ↓ in INGH; TIMP2 ↑ in INGH; TIMP1 ↓ in INGH
[24], 2014	INGH	FT (Transverse fascia); spermatic veins; GSV (great saphenous vein); plasma fluid	37/15	VAR + INGH (9); VAR + CVD (6); INGH + CVD (2); VAR + CVD + INGH (5); Control: VAR (7); INGH (3); CVD (5) = 15	MMP1-2-9-12-13	ELISA; Western blot; immunoblotting	MMP1, 2, 9, 12, 13 ↑ in groups with INGH (plasma and tissue); MMP9 ↑ in group with two or three clinical conditions; MMP9 ↑ in VAR/CVD respect INGH
[25], 2011	DIINGH, IINGH, IH, CONTROLS	Blood serum, FT	100/30	DIINGH (30), IINGH (30), IH (10), CONTROLS (30)	MMP 2	ELISA	MMP-2 ↑ in DINGH
[26], 2021	DINGH	TF (Transverse fascia)	90/30	DINGH (30); IH (30); controls (30)	TIMP1; TIMP2	Immunohistochemical	TIMP1 ↑ in DINGH and in IH; TIMP2 ↓ in DINGH

## Data Availability

Not applicable.
